# Process-Induced Morphology of Poly(Butylene Adipate Terephthalate)/Poly(Lactic Acid) Blown Extrusion Films Modified with Chain-Extending Cross-Linkers

**DOI:** 10.3390/polym14101939

**Published:** 2022-05-10

**Authors:** Juliana V. C. Azevedo, Esther Ramakers-van Dorp, Roman Grimmig, Berenika Hausnerova, Bernhard Möginger

**Affiliations:** 1 Faculty of Technology, Tomas Bata University in Zlín, Vavreckova 275, 760 01 Zlín, Czech Republic; juliana.azevedo@bio-fed.com; 2 Department of Natural Sciences, University of Applied Sciences Bonn-Rhein-Sieg, von Liebig Str. 20, 53359 Rheinbach, Germany; esther.vandorp@h-brs.de (E.R.-v.D.); roman.grimmig@h-brs.de (R.G.); bernhard.moeginger@h-brs.de (B.M.); 3 BIO-FED, Branch of AKRO-PLASTIC GmbH, BioCampus Cologne, Nattermannallee 1, 50829 Köln, Germany; 4 Centre of Polymer Systems, University Institute, Tomas Bata University in Zlín, Nam. T.G. Masaryka 5555, 760 01 Zlín, Czech Republic

**Keywords:** poly(butylene adipate terephthalate), poly(lactic acid), chain-extending cross-linker, process-induced morphology, blown film extrusion

## Abstract

Process-induced changes in the morphology of biodegradable polybutylene adipate terephthalate (PBAT) and polylactic acid (PLA) blends modified with various multifunctional chain-extending cross-linkers (CECLs) are presented. The morphology of unmodified and modified films produced with blown film extrusion is examined in an extrusion direction (ED) and a transverse direction (TD). While FTIR analysis showed only small peak shifts indicating that the CECLs modify the molecular weight of the PBAT/PLA blend, SEM investigations of the fracture surfaces of blown extrusion films revealed their significant effect on the morphology formed during the processing. Due to the combined shear and elongation deformation during blown film extrusion, rather spherical PLA islands were partly transformed into long fibrils, which tended to decay to chains of elliptical islands if cooled slowly. The CECL introduction into the blend changed the thickness of the PLA fibrils, modified the interface adhesion, and altered the deformation behavior of the PBAT matrix from brittle to ductile. The results proved that CECLs react selectively with PBAT, PLA, and their interface. Furthermore, the reactions of CECLs with PBAT/PLA induced by the processing depended on the deformation directions (ED and TD), thus resulting in further non-uniformities of blown extrusion films.

## 1. Introduction

The largest market in the plastics industry is the packaging segment, with more than 40% of plastics demand in Europe [1]. Fifty percent of all goods are packed in plastics [1]. Blown film extrusion is the most important industrial manufacturing process of polymeric films [2,3]. In this process, a molten polymer is extruded into a tube shape and subsequently drawn by nip rollers in an extrusion direction (ED). Simultaneously, the extruded melt is blown by injecting air to a substantially larger tube ratio [4,5,6,7]. As the polymer melt is drawn in both the extrusion and transverse directions (TD), the film blowing process represents a biaxial elongational flow process. The orientation of the macromolecules and the final morphology of blown films depend strongly on the chosen process parameters [2,3,4,5,6,7].

The majority of biodegradable blends are based on polybutylene adipate terephthalate (PBAT) and polylactic acid (PLA) compounds [8,9,10,11]. Their chemical reactivity is typically governed by ester, amide, and ether functional groups. PBAT is a random copolymer of butylene adipate and terephthalate, which owes its biodegradability to the butylene adipate groups and its stability and mechanical strength to the terephthalate groups [11,12,13,14]. PLA is biodegradable and entirely renewable if it originates from starch [15,16,17,18]. It often exhibits a brittle behavior, and therefore it is inappropriate for applications requiring high deformation strains [19,20]. Therefore, blending with ductile polymers such as PBAT is a reasonable approach.

Investigations on PBAT/PLA blends showed that the interfacial compatibility between PLA and PBAT is poor but can be improved by compatibilizers [21,22]. Recent developments showed that chain-extending cross-linkers (CECL) might increase melt strength, thermal stability, and phase compatibility of noncompatible polymer blends. However, only a few investigations deal with CECLs’ influence on PBAT/PLA blends [16,21,23,24,25,26,27].

Chiu et al. [16] showed on injection molded PBAT/PLA blends that the annealed PLA has a brittle and low-deformed breaking structure, and break tracks presented a considerable acute angle. The PBAT also exhibited a brittle break, but its break angle was alleviative, and its break tracks were longer than for the PLA. The break cross-sections of PBAT/PLA (30/70) and PBAT/PLA (50/50) were similar to those of PLA. The PBAT/PLA (30/70) presented an irregular layer break cross-section with PLA spheroid-dispersed in the PBAT continuous phase, and the PBAT/PLA (50/50) showed a directive-layer break cross-section. 

Wang et al. [21] found that an epoxy-terminated branched polymer (ETBP) enhances the interfacial compatibility and mechanical properties of PBAT/PLA compounds. PBAT was dispersed in the continuous PLA phase in droplet form. The phase separation structure between PLA and PBAT could be seen. The interface image between the two phases was clear and loosely bonded, showing a sea-island structure. The average size of PBAT particles in PBAT/PLA blends was 2.87 μm, indicating a typical thermodynamically immiscible system. After the addition of ETBP, the size of the dispersed PBAT particles decreased (the average size reduced by up to 0.38 μm). Moreover, the interface between PLA and PBAT became fuzzy as more PLA and PBAT were combined together. With the increase of ETBP, the tensile fracture surface became ductile, and the sea island structure nearly disappeared, indicating that the addition of more than 1.0 phr of ETBP can significantly improve the compatibility between PLA and PBAT as well as the toughness of PBAT/PLA blends [21].

Al-Itry et al. [23] modified PBAT/PLA blends with CECLs (Joncryl^®^, BASF, Germany) and confirmed improved thermal stability, increased molecular weight, intrinsic viscosity, and elastic modulus of the PBAT/PLA. 

Dong et al. [24] investigated PBAT/PLA blends with and without two chain-extending cross-linkers. SEM analysis of the reference PBAT/PLA (20/80) blend showed that the PBAT was dispersed non-uniformly in the PLA matrix with the large domain size (1~5 µm), while adhesion between the PLA and PBAT phases was poor, as evidenced by interfacial debonding and oval cavities left by the PBAT domains after cryo-fracture. The dispersion of the PBAT domains became uniform, and the average PBAT domain size was reduced to approximately 0.5 and 1 µm after the addition of 1 wt.% of Joncryl^®^ and 1,6-hexanediol diglycidyl ether, respectively. The interfacial adhesion between the PLA and PBAT phases improved, and results indicated that the compatibility between the PLA and PBAT was greatly enhanced by the incorporation of both CECL, which reasonably affected other properties of the blends.

To the best of our knowledge, only Arruda et al. [25] investigated PBAT/PLA blends modified with Joncryl^®^ on the films produced via blown film extrusion. Regarding the films without chain extenders, the PLA dispersed phase presented itself as an elongated and fibrillar structure preferably arranged towards the drawn direction of the film. Arruda et al. [25] assumed that this fibrillar morphology was caused by the elongational strain derived from the film drawing process. In the films containing 0.3 and 0.6% Joncryl^®^, the dispersed phase appeared as ellipsoids oriented towards the film drawing. The CECLs were expected to produce the PBAT/PLA copolymer.

Pan et al. [26] studied PBAT/PLA melt compounded with methylene diphenyl diisocyanate as CECL. SEM micrographs showed that when the dispersed phase concentration reached a 1:1 ratio, complex structures, such as platelet, ribbon- or sheet-like, stratified, and co-continuous, were formed. The PBAT with a size of approximately 10 mm showed almost no wetting, with the PLA phase indicating very low compatibility of the reference blend. By the formation of a PBAT/PLA copolymer due to the addition of CECL, a decrease in the size of the PBAT phase was attained.

Phetwarotai et al. [27] investigated PLA grafted with maleic anhydride (PLA-g-MA) synthesized via reactive maleation and PBAT/PLA compounds compatibilized with toluene diisocyanate (TDI). Fracture surfaces after the tension of compression-molded films exhibited poor interfacial adhesion between PLA and PBAT phases. Upon the addition of TDI, SEM showed many elongated fibrils as the addition of the TDI enabled the strong formation of urethane and/or amide linkages between PLA and PBAT phases, which improved interfacial adhesion. Further, the SEM image of the grafted blend after tension indicated the enhanced adhesion and wettability between PLA and PBAT compared to non-grafted material. The anhydride groups of PLA-g-MA could react with the hydroxyl groups of PLA and PBAT to form the ester linkages. This strong chemical bonding was an important factor that increased the interfacial adhesion between the components.

Currently, very little is known concerning the effect of multi-functional CECLs on morphology and resulting mechanical properties of the PBAT/PLA films produced via blown film extrusion. The vast majority of the studies available were carried out on samples prepared by compression or injection molding, thus not considering the process-induced changes introduced by blown film extrusion. In our previous study [28], we showed that the chemical reactions caused by CECLs incorporation into PBAT/PLA were incomplete after compounding and that the elongation during blow film extrusion brought appropriate molecular groups into reach and thus promoted crosslinking or chain scission. Therefore, the objective of this study was to investigate in detail the effect of four CECLs on the morphology of PBAT/PLA resulting from the molding route in order to optimize their processability and usage performance.

## 2. Materials and Methods

Four chain-extending cross-linkers (1 wt.%) were compounded into a reference PBAT/PLA (REF) M·VERA^®^ B5029 [29] from BIO-FED, a branch of AKRO-PLASTIC GmbH, Köln, Germany:

V1—tris(2,4-di-tert-butylphenyl)phosphite, Songnox^TM^ 1680 (Songwon Industrial Co, Ulsan, Korea) [30];

V2—1,3-phenylenebisoxazoline, 1,3-PBO powder (Evonik, Essen, Germany) [31];

V3—aromatic polycarbodiimide, Stabaxol^®^ P110 (Lanxess, Cologne, Germany) [32];

V4—poly(4,4-dicyclohexylmethanecarbodiimide), Carbodilite^TM^ HMV-15CA (Nisshinbo, Tokyo, Japan) [33].

The REF compound contained 24% by weight of calcium carbonate particles (D50 1.2 µm, top cut 4 µm), PBAT represented the matrix, and PLA represented the dispersed phase. All ingredients were evenly mixed using a Mixaco CM 150-D (Mixaco Maschinenbau, Neuenrade, Germany) and compounded with a twin-screw extruder (FEL 26 MTS, Feddem GmbH, Sinzig, Germany) with a diameter of 32 mm and an L/D of 26, a screw speed of 260 rpm and an output rate of 20 kg h^−1^.

The 25 µm thick films were produced via blown film extrusion using an LF-400 (Labtech Engineering Company, Thailand) machine with an extrusion temperature of 165 °C and a blow-up ratio (BUR) of 1:2.5. The blown film machine had a single screw with a diameter of 25 mm and an L/D of 30. From an extrusion gap of 0.8 mm, the draw ratio was estimated to be between 12 and 14. The extrusion pressures were 240 bar for the REF blend and 290 bar (V1), 159 bar (V2), 230 bar (V4), and 313 bar (V4) for the modified compounds. Storage time to testing was 24 h at 23 °C/50% r.h.

Fourier Transform Infrared Spectroscopy (FTIR) was used to identify structural changes due to chemical reactions of the CECLs with PBAT and PLA on the compounded material (granules). IR-spectra were recorded in the wavenumber range 2000 to 600 cm^−1^ using an FTIR Microscope System (Perkin Elmer Spectrum Spotlight 200, Waltham, MA, USA) with Attenuated Total Reflectance (ATR) in continuous scan mode, a spectral resolution of 16 cm^−1^ and 15 scans averages per spectrum.

**Figure 1:**
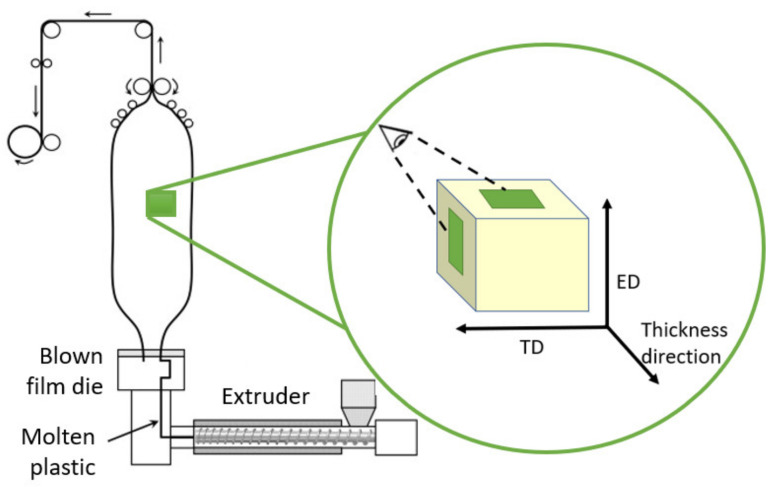
Sample orientation with respect to extrusion direction (ED), transverse direction (TD), and thickness directions; ED shows a TD-thickness direction-plain, and TD shows an ED-thickness direction-plain.

Films were fractured under cryogenic conditions in extrusion (ED) and transverse (TD) directions, as seen in Figure 1, using liquid nitrogen, and sputtered with gold at 20 mA for 3 × 30 s. Afterwards, fracture surfaces of the samples were investigated using a field-emission Scanning Electron Microscope SEM (JSM-7200F, Jeol, Tokyo, Japan) at an acceleration voltage of 5.0 kV and amplifications of 3.000 and 10.000.

## 3. Results

PBAT and PLA are immiscible polymers [34]. Their blend morphology results from the process variables (temperature, deformation types, and rates) and the properties of the components (composition, viscosity ratio, interfacial tension, continuous phase viscosity, and elasticity of the components). Due to the blown film extrusion with different draw ratios in ED and TD, anisotropic mechanical properties and corresponding differences in a morphological structure may be expected. Recently, Azevedo et al. [28] showed (based on differential scanning calorimetry data of granules and films) that the chemical reactions were incomplete after compounding and that blown film extrusion intensified them even for rapidly cooled 25 µm films. This behavior can be explained by assuming that the CECL molecules are linked with one reactive site to polymer chains during compounding, whereas the other reactive sites remain unaffected. Depending on the kind of CECL, this may also lead to chain scission. Only the chain slip due to the elongation during blown film extrusion brings appropriate molecular groups into reach. Then, the unreacted sites can react with neighboring polymer chain segments. These reactions may lead either to further chain scission or cross-linking. The fact that the elongations at the break in extrusion direction (ED) decreased with aging and remained unaltered in the transverse direction (TD) indicates that the reactions linked to chain scission and cross-linking depended also on the introduced draw ratios during film blowing, which differed for ED and TD. Furthermore, chain scission and cross-linking altered viscosity and consequently the structure, e.g., dimensions of the dispersed phase and its geometry (spherical or fibrillar). According to dynamic mechanical analysis [28], for V1 CECLs, the *T_g_* of PLA and PBAT phases were hardly affected, whereas for V2 to V4, an increase of *T_g_* in the PBAT phase and a decrease in the PLA phase were observed. This means that cross-linking mainly occurred in the PBAT phase, whereas in the PLA phase, the free volume was mainly increased by partial reaction with CECL. Finalized cross-linking in the PLA phase (with corresponding *T_g_* increases) was found for V3 and V4 compounds after the second melting.

**Figure 2:**
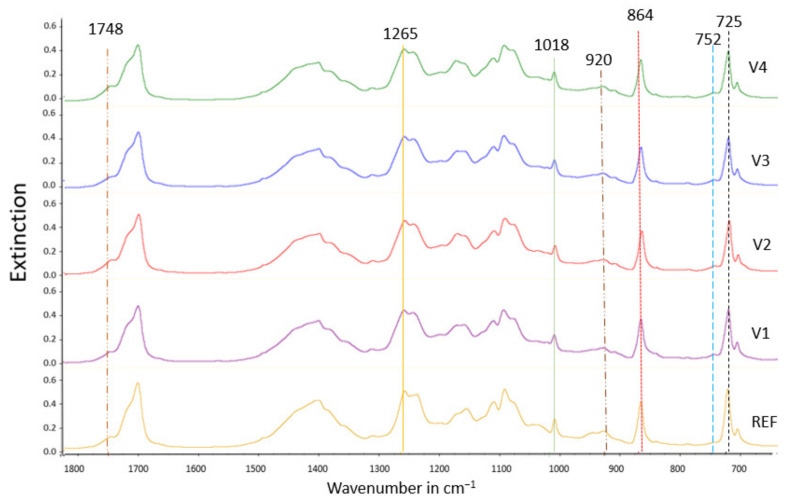
FTIR spectra of the blown films of unmodified PBAT/PLA (REF) and the CECL-modified samples (V1 to V4).

To confirm the interactions expected from DSC and mechanical analysis, FTIR-ATR was performed on the blown films (Figure 2) as well as the granules (Supplementary Materials: Figure S1). According to Standau et al. [35] and Yuniarto et al. [36], the PLA band around 752 cm^1^, together with the vibration of the α-methyl band around 864 cm^−1^, is associated with the ester (O-CH-CH_3_), while that many weaker peaks in the range of 1250-1050 cm^−1^ are assigned to C-O from carboxyl groups and C-O-C stretching vibrations, and the peak at 1748 cm^−1^ is associated with the carbonyl C=O stretching vibration.

It has been reported [23] that there are three possible linkages in polyesters: carbon-oxygen ether linkage (β-H-C hydrogen transfer), carbonyl carbon-carbon linkage, and carbonyl carbon-oxygen, which can undergo scission. The functional groups of PBAT are described as follows (Figure 2): the peak at around 1710 cm^−1^ represents carbonyl groups (C=O) in the ester linkage, while at 1265 cm^−1^, a peak intercepts C-O in the ester linkage, and at around 725 cm^−1^ a peak represents methylene (-CH2-) groups. Bending peaks of the benzene substitutes are located at wavenumbers between 700 and 900 cm^−1^.

After CECL modification of the REF blend, the PLA band at 864 cm^−1^ was slightly shifted for V2, V3, and V4 to smaller wavenumbers up to 850 cm^−1^, indicating reactions that enhance vibrations of the α-methyl group. A weak band occurring at 920 cm^−1^ is characteristic of unsaturated vinyl groups.

Overall, the spectra of V1 to V4 do not differ significantly from that obtained for REF, suggesting only small changes in the chemical structure of PBAT/PLA within the sensitivity limit of FTIR. This is in accordance with Wu et al. [37], who investigated how dicumyl peroxide (DCP) modifies the spectra of PBAT/PLA blends and found that DCP generated free radicals by thermal decomposition, initiating the formation of branching structures via hydrogen abstraction, resulting in minimal changes in the FTIR spectra. 

Furthermore, FTIR performed on the granules does not show significant differences from those obtained on blown films. Therefore, regardless of its frequent usage, FTIR may not be an efficient tool to detect the CECL-attributed chemical interactions, possibly due to the strong self-interactions of PBAT and PLA [38].

**Figure 3:**
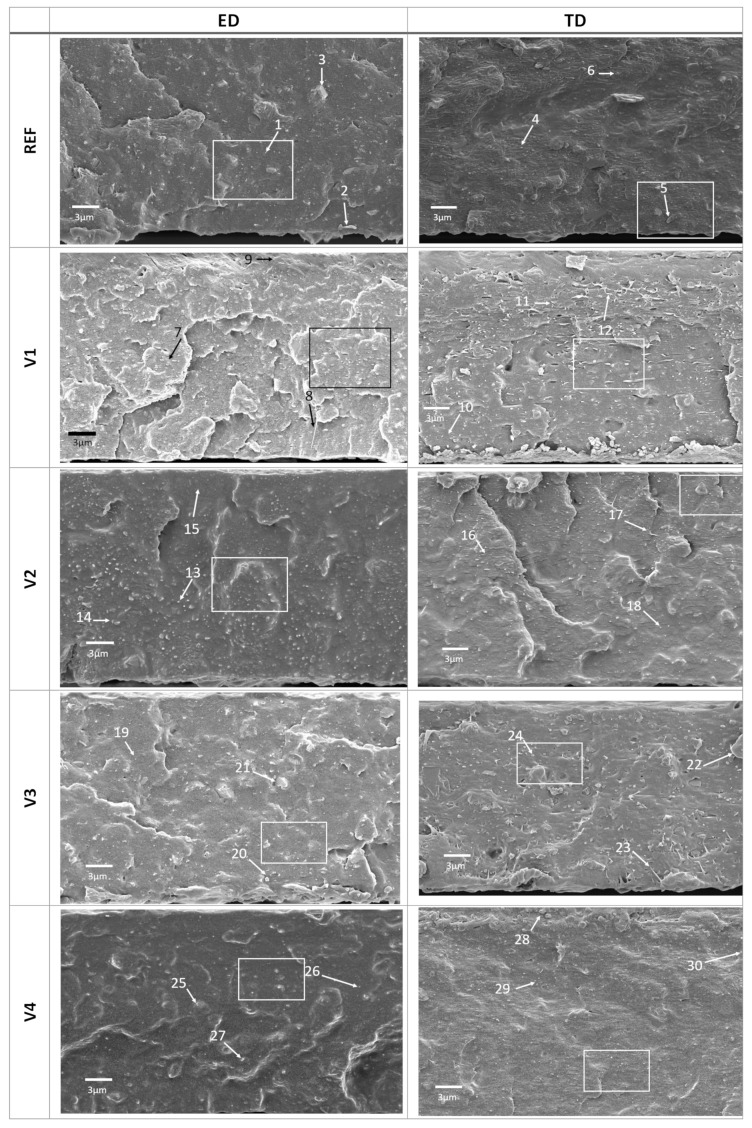
SEM of fracture surfaces of unmodified PBAT/PLA (REF) and CECL-modified (**V1 to V4**) films in ED and TD blown film extrusion directions. The rectangles represent the magnified areas displayed in Figure 4.

SEM of fracture surfaces of blown extrusion films, as seen in Figure 3, reveals that the dimensions of the dispersed PLA phase differed among the samples and depended on the orientation of the fracture surfaces.

REF blend exhibited a clear dispersion of PLA in the PBAT matrix with “sea-island” morphologies < 500 nm; see Figure 3—arrows 1 and 6, confirming nonhomogeneous dispersion reported in the literature [25,26,27]. Such morphology is associated with poor mechanical properties due to a weak interfacial adhesion between PBAT and PLA as well as internal stresses at an interface. Furthermore, the skin-core structure was found in REF consisting of coarsely dispersed PLA in the core region of the sample and fine PLA fibrils in the skin regions; see Figure 3—arrows 4 and 5 in the TD direction.

**Figure 4:**
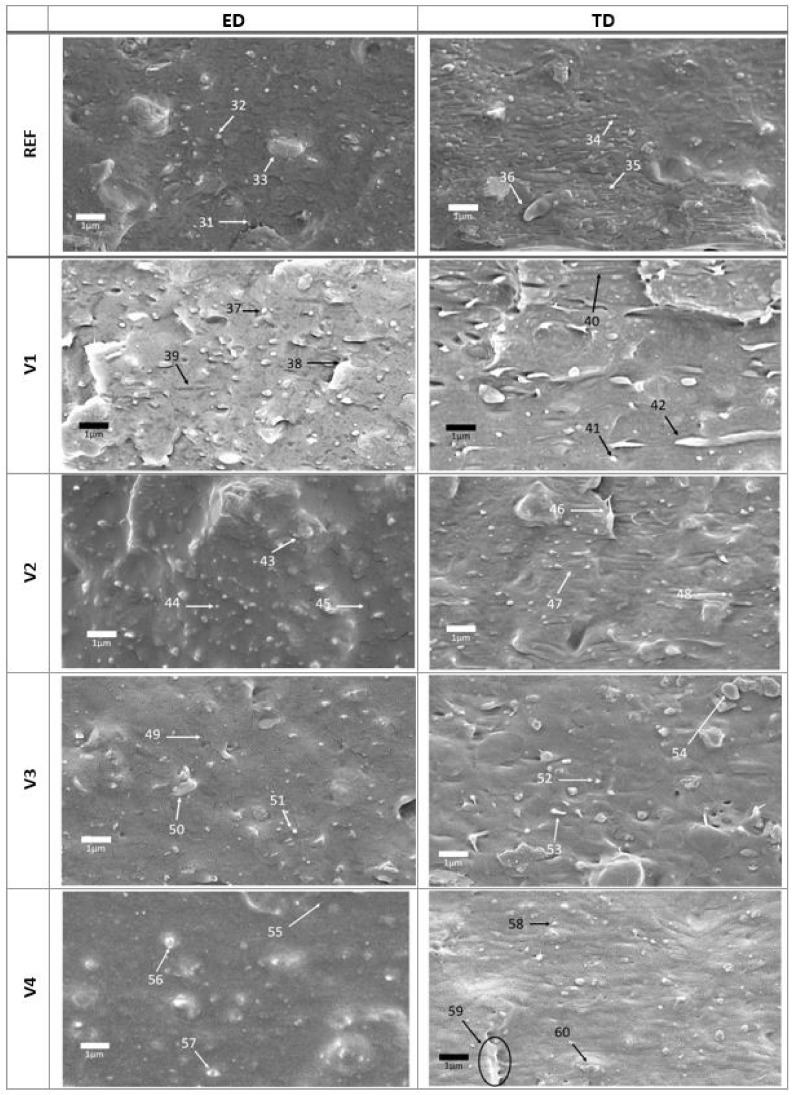
Detailed (magnification of 10,000) SEM of fracture surfaces of unmodified PBAT/PLA (REF) and CECL-modified (**V1 to V4**) films in ED and TD blown film extrusion directions.

The comparison of fracture surfaces of V1 to V4 with respect to REF shows that CECLs changed the fracture appearance as follows: V1 fails brittle, V2 slightly more ductile, and V3 to V4 significantly more ductile and tougher, as shown in Figure 3 and Figure 4. REF and V1 to V3 exhibit fibrils, as shown in Figure 3—arrows 2, 8, 12, and 23, indicating the tough and ductile behavior of the PBAT matrix).

The calcium carbonate particles having diameters of 2 to 3 µm (D50 of 1.2 µm) were well visible on the fracture surfaces, although they were completely covered by the PBAT matrix (Figure 3—arrows 3, 5, 21, and 22), indicating a good adhesion due to a complete matrix wetting, especially for V4.

The introduction of CECLs modified the morphology of the PBAT/PLA and the structure of the fracture surfaces with respect to the dimension and geometry of the PLA islands. However, there were morphological similarities in all compounds as the structure of the dispersed PLA was circular or spherical, Figure 3—arrows 1, 6, 7, 11, 13, 18, 19, 24, 28, and 30. This and the coarser islands support the process of chain scission for V1 and V2.

The differences in ED and TD were to be expected, and PLA fibrils were found only in TD (Figure 3—arrows 5, 12, 17, 23, and 28) but not in ED. In ED, the fibrils seem to be decayed in spherical or ellipsoidal PLA islands with a maximum aspect ratio of about 3, whereas in TD, both spherical PLA islands and fibrils occur. The exception is V4 modified blend, which did not exhibit PLA fibrils in TD, suggesting a better interface adhesion, where the fibrils were completely embedded in the matrix. In addition to processing-induced PLA fibrils, fracture-induced fibrils of the PBAT matrix having 5 to 10 times smaller diameters were also found (Figure 4—arrows 31, 35, 39, 40, and 46).

The morphology of the modified blends has to be interpreted also with respect to structure formation during film blowing with a BUR of 1:2.5, a drawing ratio (DR) of 12 to 14, and temperature. During the blow phase, the elongation flow stretched and oriented the melt at the beginning in TD due to blowing and subsequently in ED due to drawing. Simultaneously, the melt cooled down the faster the film was stretched and melt viscosity increased. The occurrence of the fibrils suggests that cooling and freezing exceeded the orientation relaxation.

At temperatures below 130 °C, PLA started to crystallize, freezing in the islands in the current geometry. Due to DR >> BUR, the initially spherical PLA particles in the melt were much more stretched in ED than in TD, explaining why PLA fibrils were found only in TD. After the solidification of PLA, only the PBAT matrix could be further stretched until it had been cooled below *T*_g_ at around 60 °C. During this stretching, the calcium particles are oriented perpendicular to the thickness direction.

At larger magnifications, the fibrils due to fracturing of the PBAT matrix were well visible (Figure 4—arrows 31, 35, 39, 40, and 46). This can be explained by the lower stiffness and yield stress of PBAT (*E* ≈ 400 MPa, *σ_y_* ≈ 35 MPa) [9,11] compared to PLA (*E* = 3500 MPa, *σ_y_* ≈ 60 MPa) [20], leaving the PLA in an almost nondeformed state during the fracture. Furthermore, it also enhances the fracture propagation along the PBAT/PLA interface. Rather poor adhesion is indicated with many small PLA particles (*D* < 200 nm), (Figure 4—arrows 32, 34, 37, 41, 44, 48, 51, 52, 56, and 58).

The cracks appearing on the fracture surfaces of V2 to V4 (Figure 4—arrows 45, 49, 55, and 59) were presumably not a result of the CECL modification, but of electron beam heating of the surface causing the relaxation of internal stresses and void formation.

In V1 and V3, the dispersed PLA was not covered by the PBAT matrix, indicating that the CECLs hardly affected compatibilization. The more elliptic shape of the PLA islands could have arisen due to the fact that the processing-induced liquid PLA fibrils to decay in ellipsoidal droplets, which were frozen in the current shape.

Finally, the morphological features of REF and V1 to V4, considered with respect to the degree of brittleness, structure of the PLA phase, interface adhesion of the dispersed PLA, and interface adhesion of filler particles, are summarized in Table 1.

## 4. Conclusions

The PBAT/PLA compound was modified with four multi-functional chain-extending cross-linkers (CECL). Previous calorimetric investigation of PBAT/PLA films revealed their selective reactions with the introduced CECL. The cross-linking effect occurred only for aromatic polycarbodiimide and poly(4,4-dicyclohexylmethanecarbodiimide, whereas chain scission was attained for modification with tris(2,4-di-tert-butylphenyl)phosphite and 1,3-phenylenebisoxazoline. FTIR did not prove to support the results of calorimetry and mechanical performance in a convincing way. Only slight shifts to lower wavenumbers were obtained for aromatic polycarbodiimide and poly(4,4-dicyclohexylmethanecarbodiimide, but also for 1,3-phenylenebisoxazoline. Morphological analysis was in accordance with the resulting mechanical properties. Tris(2,4-di-tert-butylphenyl)phosphite and aromatic polycarbodiimide showed a dispersed PLA phase that was not covered by the PBAT matrix, indicating that these two CECLs do not provide compatibilization, whereas poly(4,4-dicyclohexylmethanecarbodiimide) showed the dispersed PLA partially covered by the PBAT matrix. The most synergetic effect was obtained for 1,3-phenylenebisoxazoline, where the PLA phase was well embedded in the PBAT matrix, indicating adhesion and improved compatibilization.

## Figures and Tables

**Table 1:** Summary of structure features seen on fracture surfaces of unmodified PBAT/PLA (REF) and CECL-modified (V1 to V4) films in ED and TD blown molding directions.

Cpd	Feature	ED	TD
REF	brittleness	semi-brittle	semi-tough
	dispersed PLA	circular *D* ≈ 100–400 nm no fibrillar structure	circular *D* ≈ 100–400 nm partly fibrillar in ED: *D* ≈ 100 nm, *L* ≈ 1000–2000 nm
	interface adhesion of dispersed PLA	rather poor PLA surface visible, holes of PLA phase dimensions	rather poor; PLA surface visible, holes of PLA phase dimensions; lines in fracture surface indicating poor interface adhesion of fibrils
	particle adhesion	filler particles completely covered with PBAT indicating good to excellent adhesion	
**V1**	brittleness	brittle	semi-brittle
	dispersed PLA	Circular/slightly elongated *D* ≈ 100–200 nm; lines in fracture surface: *D* ≈ 150 nm, *L* ≈ 1000–1500 nm	circular *D* ≈ 100–200 nm; elongated fibrils fibrils: *D* ≈ 200 nm, *L* ≈ 1000–4000 nm
	Interface adhesion of dispersed PLA	bad; PLA surface visible, holes of PLA phase dimensions	bad; well visible PLA islands and fibrils; lines in fracture surface indicating bad adhesion
	particle adhesion	filler particles completely covered with PBAT indicating good to excellent adhesion	
**V2**	brittleness	ductile	tough with partly fibrillated matrix
	dispersed PLA	circular *D* ≈ 100–200 nm no fibrils	circular islands *D* ≈ 100–200 nm elongated fibrils: *D* ≈ 100 nm, *L* ≈ 1000–2000 nm
	interface adhesion of dispersed PLA	poor well embedded islands with cracks in all directions, max *L* ≈ 1000 nm	bad to poor deformed islands/fibrils in fracture surface indicating some adhesion
	matrix particle adhesion	filler particles completely covered with PBAT indicating fair to excellent adhesion	
**V3**	brittleness	ductile to tough	ductile with fibrillated matrix
	dispersed PLA	circular/slightly elliptic *D* ≈ 200–500 nm; no fibrils	circular, partly elongated *D* ≈ 200–500 nm no fibrils
	interface adhesion of dispersed PLA	bad to mean well-embedded islands with visible surface and cracks, max *L* ≈ 500 nm	bad to mean some islands partly embedded
	particle adhesion	filler particles completely covered with PBAT, indicating fair to excellent adhesion	
**V4**	brittleness	ductile to tough	poor to tough with fibrillated matrix
	dispersed PLA	circular islands *D* ≈ 100–300 nm	circular islands *D* ≈ 100–300 nm no fibrils; lines with max *L* ≈ 2500 nm
	interface adhesion of dispersed PLA	poor; PLA surface partly covered with matrix	poor; no visible fibrils PLA surface partly covered with matrix
	particle adhesion	filler particles were hardly visible indicating good dispersion of the particles in the matrix	

## Data Availability

Data are stored at the personal depository of J. Azevedo.

## Supplementary Materials

The following are available online at https://www.mdpi.com/article/10.3390/polym14101939/s1, Figure S1: FTIR spectra of the granules of unmodified PBAT/PLA (REF) and the CECL-modified samples (V1 to V4).
