# Supplementary material for: Process-Induced Morphology of Poly(Butylene Adipate Terephthalate)/Poly(Lactic Acid) Blown Extrusion Films Modified with Chain-Extending Cross-Linkers
Source: Polymers (Basel). 2022 May 10;14(10):1939. doi: 10.3390/polym14101939 (PMC9144630; doi:10.3390/polym14101939)
Supplement: Supplementary file 1 [file polymers-14-01939-s001.zip › polymers-1711239-supplementary.pdf]

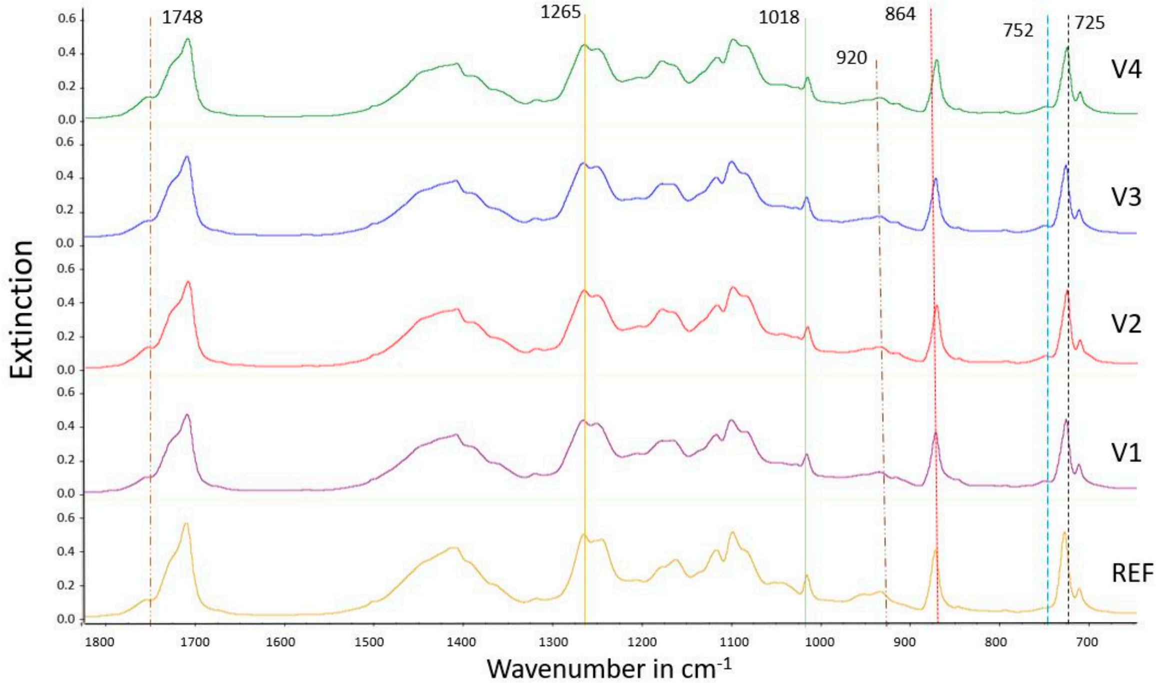

Figure S1. FTIR spectra of the granules of unmodified PBAT/PLA (REF) and the CECL-modified samples (V1 to V4).
